# Extending the EQ-5D: the case for a complementary set of 4 psycho-social dimensions

**DOI:** 10.1007/s11136-022-03243-7

**Published:** 2022-09-20

**Authors:** Gang Chen, Jan Abel Olsen

**Affiliations:** 1 Centre for Health Economics, Monash Business School, Monash University, Victoria, 3145 Australia; 2 Department of Community Medicine, UiT—the Arctic University of Norway, 9037 Tromsø, Norway; 3 Division of Health Services, Norwegian Institute of Public Health, 0213 Oslo, Norway

**Keywords:** Health-related quality of life, Subjective well-being, Health utility, EQ-5D, Bolt-on

## Abstract

**Objectives:**

The EQ-5D is the most widely applied preference-based health-related quality of life measure. However, concerns have been raised that the existing dimensional structure lacks sufficient components of mental and social aspects of health. This study empirically explored the performance of a coherent set of four psycho-social bolt-ons: Vitality; Sleep; Personal relationships; and Social isolation.

**Methods:**

Cross-sectional surveys were conducted with online panel members from five countries (Australia, Canada, Norway, UK, US) (total *N* = 4786). Four bolt-ons were described using terms aligned with EQ nomenclature. Latent structures among all nine dimensions are studied using an exploratory factor analysis (EFA). The Shorrocks-Shapely decomposition analyses are conducted to illustrate the relative importance of the nine dimensions in explaining two outcome measures for health (EQ-VAS, satisfaction with health) and two for subjective well-being (the hedonic approach of global life satisfaction and an eudemonic item on meaningfulness). Sub-group analyses are performed on older adults (65 +) and socially disadvantaged groups.

**Results:**

Strength of correlations among four bolt-ons ranges from 0.34 to 0.49. As for their correlations with the EQ-5D dimensions, they are generally much less correlated with four physical health dimensions than with mental health dimensions (ranged from 0.21 to 0.50). The EFA identifies two latent factors. When explaining health, *Vitality* is the most important. When explaining subjective well-being, *Social isolation* is second most important, after Anxiety/depression.

**Conclusion:**

We provide evidence that further complementing the current EQ-5D-5L health state classification system with a coherent set of four bolt-on dimensions that will fill its psycho-social gap.

**Supplementary Information:**

The online version contains supplementary material available at 10.1007/s11136-022-03243-7.

## Introduction

The EQ-5D is by far the most widely applied generic preference-based measure (GPBM) of health-related quality of life (HRQoL). Reviews of economic evaluations using GPBMs confirm its dominant position: the EQ-5D was applied in 63% of those published in the period (2005–2010) [1] and 77% of those published in 2010 only [2]. In several countries across the globe, its application has now expanded beyond economic evaluations to include clinical studies, national quality registries, and population health surveys (www.euroqol.org).

Over the last decade, the instrument has been extended ‘level-wise,’ from a three-level to a five-level descriptive system [3], something that has initiated immense research activity on estimating country-specific value sets. However, concerns have been raised that the existing dimensional structure does not sufficiently capture important mental and social aspects of health, i.e., that the descriptive system should be extended ‘dimension-wise’ [4–6]. Relevant to this literature, empirical studies that directly compare EQ-5D and subjective well-being (SWB) measures have highlighted a complementary role between them [7–10] and indicate the need to integrate these measures in clinical practice and healthcare evaluation, a suggestion that has also attained from literature not focusing on preference-based HRQoL [11]. Richardson et al. [12] and Chen et al. [13] revealed that having a classification system with more psycho-social components could be a solution. For example, when empirically examining the relationships between a capability well-being measure (Investigating Choice Experiments Capability Measure—Adults, ICECAP-A) and two alternative GPBMs (EQ-5D-5L and Assessment of Quality of Life, AQoL-8D), Chen et al. [13] found that the complementary relationship between ICECAP-A and EQ-5D-5L was much lower than that between ICECAP-A and AQoL-8D.

In response to this concern, we have provided empirical support for adding four psycho-social ‘bolt-ons’ to the current EQ-5D-5L [14]. On the theoretical reasoning behind the choice of the four bolt-on dimensions, see the analytical framework developed by Olsen and Misajon [15]. Based on this previous work, the aim of the current study is to investigate the relative importance of a coherent set of four psycho-social bolt-ons for explaining variations in health and well-being.

The relevance of our suggested set of psycho-social dimensions (Vitality, Sleep, Personal relationships, Social isolation) has increased in a time with more public attention on how mental health, and social isolation—or loneliness—affect individuals’ quality of life and well-being [16]. A prime example from the United Kingdom (UK) in 2018 was the appointment of a ‘minister for loneliness’ to tackle social isolation. Moreover, the recent COVID-19 pandemic has made policy makers increasingly aware of the need to account for psycho-social aspects of health.

Thus, to further develop our previous work, the current paper makes three important contributions. First, the bolt-on dimensions are now described using terms aligned with the EQ nomenclature. The previous analyses were based on survey data that had included other GPBMs, from which the four dimensions were described in the AQoL-8D [14]. Second, we estimate the relative importance of the nine dimensions (including the current EQ-5D-5L) for explaining variations by the use of alternative measures of health and well-being. Third, we investigate the heterogeneity of findings by specifically exploring the results from vulnerable populations.

The paper is structured as follows: Next section presents the data and the methods. The results section firstly presents the latent structure of nine dimensions and then focuses on the relative importance of the nine dimensions for explaining variations in two different subjective health measures: a visual analogue scale (EQ-VAS) and satisfaction with personal health (from the Personal Well-being Index, PWI [17]), as well as in two well-being measures: the hedonic approach of global life satisfaction (first item in the PWI), as well as an eudemonic item of meaningfulness. Lastly, the discussion section demonstrates the potential performance of the proposed four dimensions and points to some important areas for further research.

## Methods

We use data from an anonymous survey developed on an online survey platform, Qualtrics (www.qualtrics.com). Respondents were recruited through a global company, Cint (www.cint.com), among members of its panels. For each of the five countries (Australia, Canada, Norway, the UK, and the United States (US)), demographic quotas (with regard to age and sex distribution) were applied.

Respondents from Australia and Norway were recruited in December 2018–February 2019, with a targeting sample size of 1400 in each country. The final sample consists of 1423 in Australia and 1400 in Norway (for details see Lindberg et al. [18]). Respondents were randomly selected to either of three versions of the questionnaire varying by how the bolt-on dimensions and their severity levels were described. One of the three versions applied a description of the bolt-ons that was aligned with the EQ nomenclature (*N* = 472 in Australia and *N* = 464 in Norway). After analyzing the data, we decided to only use this version (see Appendix) when the survey was rolled out in three more countries.

Respondents from Canada, the UK, and the US were recruited in April 2020, and the targeting sample size was 1200 in each country. Initially, a total of 1459 respondents each in Canada and the UK and 1517 in the US consented and clicked the survey link. Next, respondents were excluded if they (a) did not submit the survey, or the quota was full (*N* = 172 in Canada; *N* = 168 in the UK; *N* = 230 in the US), or (b) failed quality thresholds, e.g., spent less than the defined minimum time^1^ to complete the survey (*N* = 9 in Canada; *N* = 3 in the UK; *N* = 3 in the US). After the exclusion, the Canadian, the UK and the US sample sizes were left at *N* = 1278, *N* = 1288, and *N* = 1284, respectively.

### Variables

The EQ-5D-5L descriptive system consists of five dimensions (Mobility, Self-care, Usual activities, Pain/discomfort, Anxiety/depression), each with five severity levels. Thereafter, we included four additional dimensions (Vitality, Sleep, Personal relationships, and Social isolation), each described using *four* levels: no problems, slight, moderate, and severe. The fifth level for ‘unable’/‘extreme’ was disregarded because (i) there is evidence that respondents have difficulties distinguishing the relative severity between levels 4 and 5 in the EQ-5D-5L descriptive system and (ii) the proportion of respondents ticking ‘extreme’ is generally extremely low [19, 20]. As for the current context of psycho-social domains, we do not see any a priori reasons why distinguishing between severe and extreme would be less difficult or that the prevalence of ‘extreme’ would be higher. Thus, our suggested **p**sycho-**s**ocial bolt-on to the EQ-5D-5L could be denoted PS-4D-4L. After describing their health, respondents were presented with the EQ-VAS to rate their overall HRQoL on a vertical scale [0–100] (the worst health you can imagine—the best health you can imagine).

Prior to the tasks of describing and valuing their health, respondents answered the PWI where the domains are rated on a horizontal scale [0–10] (not at all—completely). For the current paper, we use the health domain item (‘How satisfied are you with your health?’) and the evaluative global life satisfaction (GLS) item (‘Thinking about your own life and personal circumstances, how satisfied are you with your life as a whole?). In addition, we included one item to measure the eudemonic part of SWB (‘Overall, to what extent do you feel the things you do in your life are meaningful?’), which is rated on a [0–10] (not at all—completely) scale. Thus, by considering both an evaluative and a eudemonic conception of well-being we provide a more comprehensive picture of how these nine dimensions are associated with multi-dimensional well-being. The choice of focusing on the above two items, life satisfaction and worthwhile activity, is consistent with the recommendation from VanderWeele et al. [21], based on the evidence that “these two items have been used extensively, have broad conceptual coverage, …, show some of the highest and most consistent correlations with much broader well-being measures (page 3).”

Demographics and socio-economic characteristics (such as age, gender, education levels, gross household income) were asked at the end of the questionnaire, including the widely used MacArthur scale of subjective social status [22]. Originally developed for the US, respondents are presented a ladder with 10 steps and the instruction: “Think of the ladder as representing where people stand in society. At the top of the ladder are the people who are best off—those who have the most money, education, and the best jobs. At the bottom are the people who are worst off—those who have the least money, least education, and the worst jobs or no job. The higher up you are on this ladder, the closer you are to people at the very top and the lower you are, the closer you are to the bottom.”

*Subjective* social status represents a different concept from the more *objective* indicators of socio-economic position, such as education and income (see Lindberg et al. [18]). Thus, it is included in separate analyses to check the robustness of the findings.

We investigate three groups of *vulnerable populations*: (i) elderly (65 years and above); (ii) low subjective social status (score 4 or below on the MacArthur ladder, representing around 25% of respondents in each country); and (iii) low socio-economic position, defined by a combination of low education (non-tertiary) and relatively lower gross household income,^2^ such that they account for less than 20% of total respondents in each country.

### Statistical analyses

The statistical analyses aim to show the performance of the four bolt-on dimensions. First, descriptive statistics are used to explore to what extent the inclusion of the bolt-ons would reduce the ceiling effect in EQ-5D-5L (i.e., respondents with level 1 on all five dimensions). Next, Spearman’s rank correlation coefficient and exploratory factor analysis (EFA) were used to explore the latent structure among the nine dimensions. Considering the additional unique contribution of the bolt-on dimensions to the existing EQ-5D-5L classification system, it is hypothesized that the bolt-on dimensions should not be strongly correlated (according to a commonly adopted threshold of 0.7 [9, 10]) among themselves or with existing EQ-5D-5L dimensions. The EFA is conducted to reveal the latent factor structures among the nine items. The EFA is estimated using the maximum likelihood method, while the number of factors is extracted based on the minimum average partial method [23, 24]. Taking into account the potential correlations among factors, we report the pattern matrix from the rotated results by the oblique Promax method.

Next, we reveal the contributions that the nine dimensions have for explaining variations in health and well-being, respectively. In the regression framework, several methods could potentially be used to understand the relative importance of the independent variables, such as calculating the standardized regression coefficients [9, 10]. This approach may be convenient if each dimension is included as a continuous variable. However, in our study, each dimension was coded as a set of dummy variables. To illustrate the relative importance of the nine dimensions, we apply the Shorrocks-Shapely decomposition analysis (with regard to the R-squared statistics post the ordinary least squares regression) [25, 26]. This variance decomposition technique measures the marginal contribution to the regression model’s explained variance which would facilitate an easy interpretation of the relative contribution from each of the nine dimensions. It also copes with the existence of potential multicollinearity of independent variables [27].

Separate regressions are run on four dependent variables: two health measures and two well-being measures. In a general public sample, few respondents report more impaired levels of physical health-related dimensions, in particular, Self-care and Mobility (as can be seen in the results). In the model specification presented in the table, we handle the inconsistency of the regression coefficients by combining the more severe levels within each EQ-5D-5L dimension based on the equation, while EQ-VAS was the dependent variable.

Except for EFA which was conducted using EViews version 11 (IHS Global Inc., Irvine, CA, USA), all other analyses were conducted using Stata version 16 (StataCorp LP, College Station, Texas, USA).

## Results

Table 1 presents the sample characteristics. For the pooled sample (*N* = 4786), they had a mean (standard deviation) age of 45 (16) years. Around 50% are females and around 49% had education attainment below a bachelor’s degree.

**Table 1:** Respondents’ characteristics

	Pooled
	*N* = 4786
Panel A: Socio-demographic characteristics, %	
Gender	
Female	49.9
Age (years)	
18–24	12.4
25–44	37.8
45–64	34.7
65 +	15.0
Education	
High school or below	21.1
Certificate/Diploma	28.2
Bachelor degree	32.9
Postgraduate degree	17.9
Gross household income	
Low	30.6
Socio-economic position	
Deprived (low household income & non-tertiary education)	18.2
MacArthur scale of subjective social status [1–10]	
Mean (standard deviation)	5.8 (2.0)
Low (1–4)	23.5
Country	
Australia	9.9
Canada	26.7
Norway	9.7
The UK	26.9
The USA	26.8
Panel B: health & well-being, mean (standard deviation)	
EQ-VAS	73.7 (20.2)
Satisfaction with health	6.8 (2.3)
Global life satisfaction (GLS)	6.8 (2.2)
Meaningful life	6.9 (2.3)

Table 2 shows the proportion of responses to each of the nine dimensions. Respondents were most likely to report having any degree of impairments in Sleep (63.6%) and Vitality (60.2%). On the contrary, Self-care (13.2%) was the dimension that respondents were least likely to report having any impairments.

**Table 2:** The proportion of responses to nine health-related quality of life dimensions, %

Levels	Mobility	Self-care	Usual activities	Pain/discomfort	Anxiety/depression	Vitality	Sleep	Personal relationships	Social isolation
1	74.7	86.8	72.3	43.7	42.5	39.8	36.4	56.7	54.8
2	14.1	7.4	15.4	35.1	29.7	39.7	37.2	28.9	27.0
3	7.5	3.7	8.2	14.9	17.3	15.2	18.9	10.7	12.4
4	2.8	1.6	3.0	4.8	6.5	5.2	7.4	3.7	5.8
5	1.0	0.6	1.1	1.5	4.1	N/A	N/A	N/A	N/A

*N* = 4786

*N/A* not applicable

When focusing on those who had ‘full health’ according to the EQ-5D-5L classification system (*N* = 1125), Table 3 shows that around 27.7% of these respondents reported having any impairments with Sleep, 18.1% with Vitality, 15.4% with Personal relationships, and 14.7% with Social isolation.

**Table 3:** The proportion of responses to four bolt-on dimensions when EQ-5D-5L equals 1, %

Levels	Vitality	Sleep	Personal relationships	Social isolation
1	81.9	72.3	84.6	85.3
2	16.4	24.1	12.4	11.6
3	1.2	3.4	2.4	2.4
4	0.4	0.3	0.6	0.6

*N* = 1125

Examining the strength of correlations among nine dimensions, Table 4 shows the highest correlations between Mobility and Usual activities (r = 0.65) and the lowest between Mobility and Social isolation (r = 0.21) and between Mobility and Personal relationships (0.21). Among the four bolt-ons, the correlations varied between 0.34 and 0.49. As for their correlations with the EQ-5D dimensions, they were generally much less correlated with the physical health dimensions than with the mental health dimension Anxiety/depression (ranged from 0.21 to 0.50). For the subsample of those classified as ‘full health’ based on EQ-5D-5L, the correlation coefficients among the four bolt-on dimensions ranged from 0.19 (Vitality and Personal relationships) to 0.28 (Vitality and Sleep), further supporting evidence of non-overlap across the four bolt-on dimensions (Online Supplementary Table 1). The EFA results showed that two factors were extracted from nine dimensions; in particular, the four proposed bolt-on dimensions and the Anxiety/Depression from EQ-5D-5L were grouped together to represent psycho-social health (Online Supplementary Table 2).

**Table 4:** Correlations among health-related quality of life dimensions (*N* = 4786)

	Mobility	Self-care	Usual activities	Pain/discomfort	Anxiety/depression	Vitality	Sleep	Personal relationships	Social isolation
Mobility	1								
Self-care	0.529	1							
Usual activities	0.650	0.544	1						
Pain/discomfort	0.542	0.366	0.532	1					
Anxiety/depression	0.238	0.257	0.336	0.327	1				
Vitality	0.385	0.288	0.440	0.454	0.489	1			
Sleep	0.294	0.247	0.354	0.396	0.481	0.490	1		
Personal relationships	0.210	0.238	0.280	0.256	0.461	0.357	0.344	1	
Social isolation	0.207	0.240	0.304	0.243	0.503	0.405	0.361	0.475	1

All Spearman correlation coefficients were statistically significant (all *p*-value < 0.01)

Table 5 presents four sets of regression coefficients corresponding to four outcomes. We have combined more severe levels within each EQ-5D-5L dimension with the aim to cope with some inconsistent raw coefficients from the EQ-VAS equation (not reported). For example, the third to fifth levels of Mobility dimension were combined and the second to fifth levels of the Self-care dimension were combined. However, the inconsistency of Self-care dimension remains, though insignificant. As for the two equations on SWB (last two columns), it appears that all four physical health dimensions in the EQ-5D were insignificant or inconsistent (Self-care). Thus, what explains SWB was mainly Anxiety/Depression and the four bolt-on dimensions.

**Table 5:** Experience weighting on EQ-5D plus bolt-on dimensions

Dimensions	Levels	EQ-VAS	Satisfaction: health	Satisfaction: life as a whole	Meaningful life
Mobility	2	−3.187	−0.467	0.029	0.084
		(0.812)**	(0.097)**	(0.097)	(0.102)
	3, 4 & 5	−6.303	−0.336	0.236	0.189
		(1.097)**	(0.131)*	(0.131)	(0.138)
Self-care	2, 3, 4 & 5	0.252	0.612	0.685	0.666
		(0.926)	(0.110)**	(0.110)**	(0.117)**
Usual activities	2	−2.548	−0.462	−0.071	0.001
		(0.795)**	(0.095)**	(0.095)	(0.100)
	3	−4.024	−0.781	−0.439	−0.348
		(1.147)**	(0.137)**	(0.137)**	(0.144)*
	4 & 5	−4.588	−0.945	−0.207	−0.161
		(1.644)**	(0.196)**	(0.196)	(0.207)
Pain/discomfort	2	−1.199	−0.264	0.150	0.072
		(0.592)*	(0.071)**	(0.071)*	(0.075)
	3	−6.248	−0.770	0.024	0.073
		(0.880)**	(0.105)**	(0.105)	(0.111)
	4 & 5	−9.352	−0.631	0.296	0.530
		(1.321)**	(0.157)**	(0.157)	(0.166)**
Anxiety/depression	2	−1.492	−0.276	−0.521	−0.355
		(0.633)*	(0.075)**	(0.075)**	(0.080)**
	3	−2.726	−0.390	−0.854	−0.618
		(0.811)**	(0.097)**	(0.097)**	(0.102)**
	4 & 5	−6.640	−0.615	−1.476	−1.098
		(1.063)**	(0.127)**	(0.127)**	(0.134)**
Vitality	2	−5.299	−0.706	−0.392	−0.364
		(0.606)**	(0.072)**	(0.072)**	(0.076)**
	3	−10.469	−1.421	−0.758	−0.931
		(0.872)**	(0.104)**	(0.104)**	(0.110)**
	4	−18.625	−2.008	−0.903	−1.273
		(1.348)**	(0.161)**	(0.161)**	(0.170)**
Sleep	2	−1.078	−0.122	−0.068	−0.130
		(0.595)	(0.071)	(0.071)	(0.075)
	3	−2.285	−0.215	0.002	−0.114
		(0.783)**	(0.093)*	(0.093)	(0.099)
	4	−4.158	−0.371	−0.358	−0.216
		(1.124)**	(0.134)**	(0.134)**	(0.142)
Personal relationships	2	−0.652	−0.043	−0.447	−0.443
		(0.593)	(0.071)	(0.071)**	(0.075)**
	3	−1.162	−0.117	−0.849	−0.932
		(0.890)	(0.106)	(0.106)**	(0.112)**
	4	−2.793	0.002	−1.021	−0.953
		(1.434)	(0.171)	(0.171)**	(0.181)**
Social isolation	2	−0.987	−0.095	−0.206	−0.296
		(0.610)	(0.073)	(0.073)**	(0.077)**
	3	−2.008	−0.115	−0.495	−0.506
		(0.852)*	(0.101)	(0.102)**	(0.107)**
	4	−5.453	−0.707	−1.303	−1.484
		(1.234)**	(0.147)**	(0.147)**	(0.155)**
Age & Gender dummies		√	√	√	√
Education dummy		√	√	√	√
Country dummies		√	√	√	√
Observations		4786	4786	4786	4786
R-squared		0.357	0.305	0.253	0.226

Standard errors in parentheses. **p < 0.01, *p < 0.05

Overall the nine dimensions explained 35.7% of the total variance of EQ-VAS, which was higher than the explained variance of the health satisfaction item from the PWI (30.5%). As for the variance in SWB, the nine dimensions explained 25.3% of the GLS, which was higher than the explained variance in the eudemonic item (22.6%). Thus, for the following subgroup analyses, we concentrate on EQ-VAS as our health outcome and GLS as our SWB outcome.

**Fig. 1:**
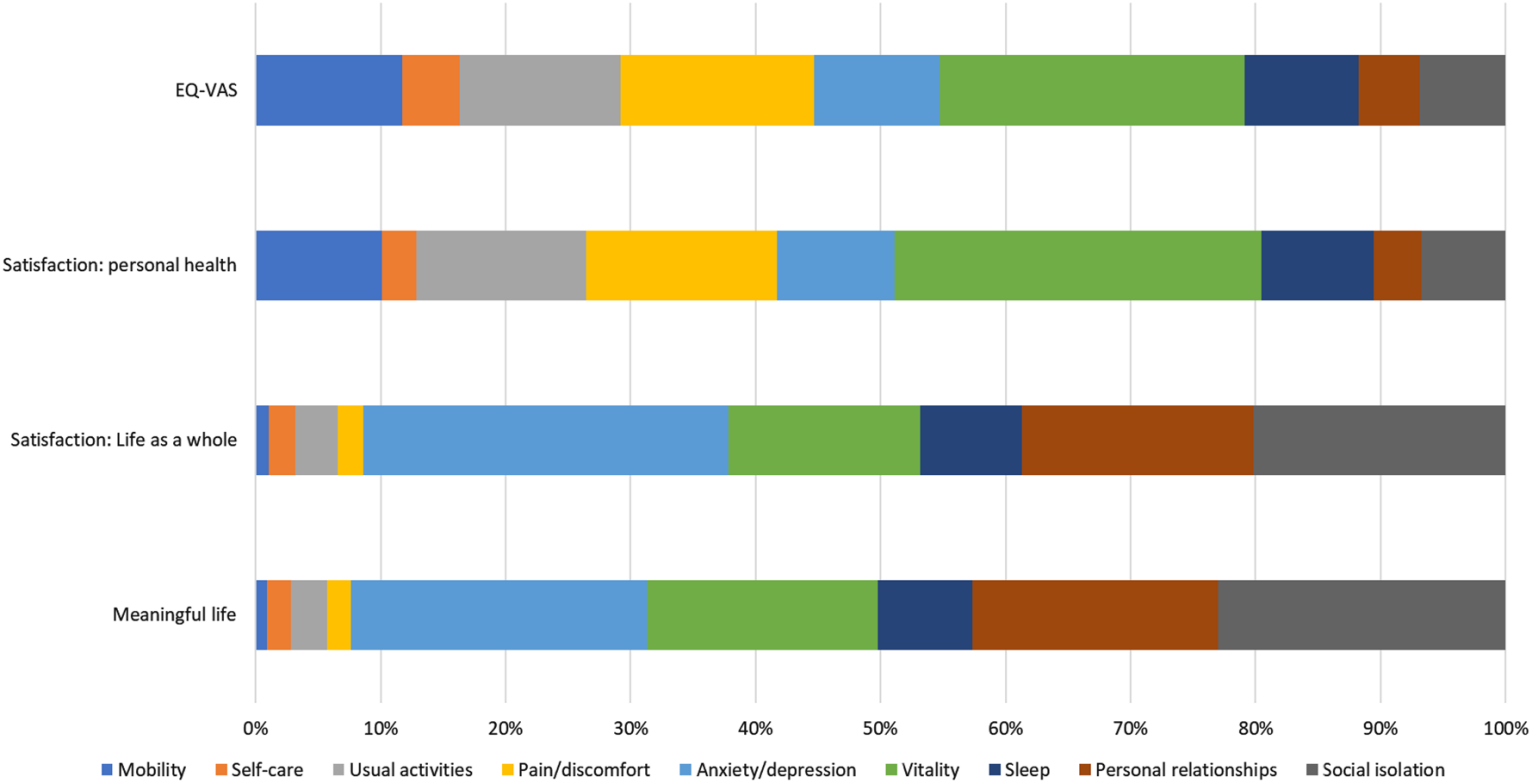
Shorrocks-Shapely decomposition results by outcomes

Figure 1 reported the Shorrocks-Shapely decomposition results which show the relative importance of each dimension. With EQ-VAS as the dependent variable, Vitality was clearly the most important dimension, followed by Pain/Discomfort, Usual activities, Mobility, Anxiety/Depression, Sleep, Social isolation, Personal relationships, and Self-care. A very similar pattern and identical ranking were observed when satisfaction with personal health was used as the dependent variable.

When considering SWB, Fig. 1 shows similar patterns across the two outcome measures on the relative importance of the nine dimensions. Note that the four physical health dimensions in the EQ-5D-5L explain less than 9% of the total explained variance.

Among the four bolt-on dimensions, Vitality was the most important for health, while the two items on social relationships were most important in explaining variations in SWB, 40% of the total explained variance.

**Fig. 2:**
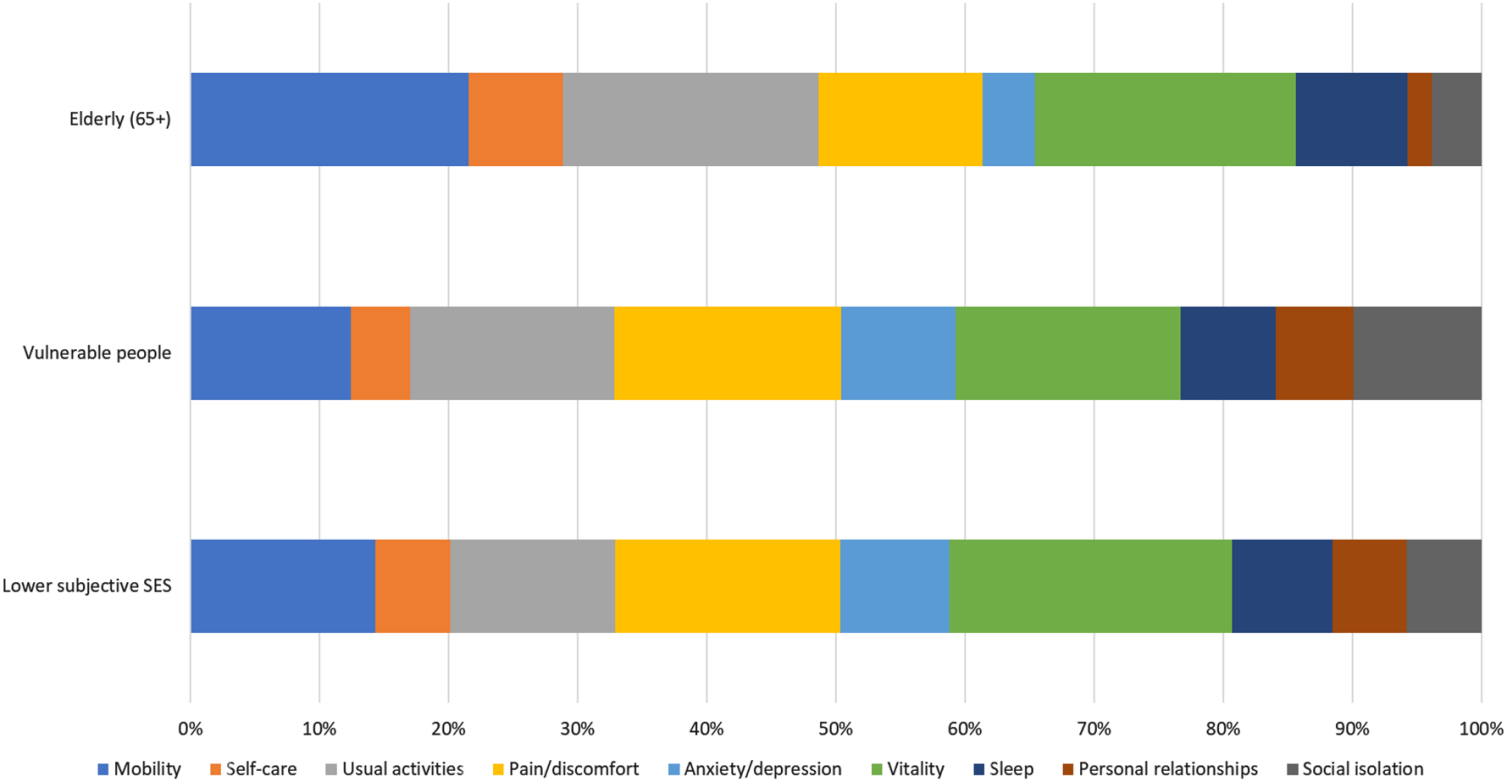
Shorrocks-Shapely decomposition results of EQ-VAS, subgroup analyses results. Elderly (aged 65 and over), 15% total sample. Vulnerable people (lower educated & lower household income), 18% total sample. Lower subjective SES (scored 4 or lower on the MacArthur ladder), 23% total sample

Figure 2 reports subgroups’ results on health. In the elderly group, the four bolt-on dimensions explain less of the total variation than for the full sample in Fig. 1 (35% vs 45%), something that is understandable given the increased impairment in physical function associated with older age: the three dimensions Mobility, Self-care and Usual activities explain as much as 48% in the elderly subgroup, as compared to 28% in the full sample in Fig. 1. In the deprived socio-economic subgroup, the higher importance of the four physical health dimensions (as compared to the full sample) would reflect that their health is generally worse. Interestingly, our two items on social relationships were more important for health in this deprived subgroup than for the full sample.

**Fig. 3:**
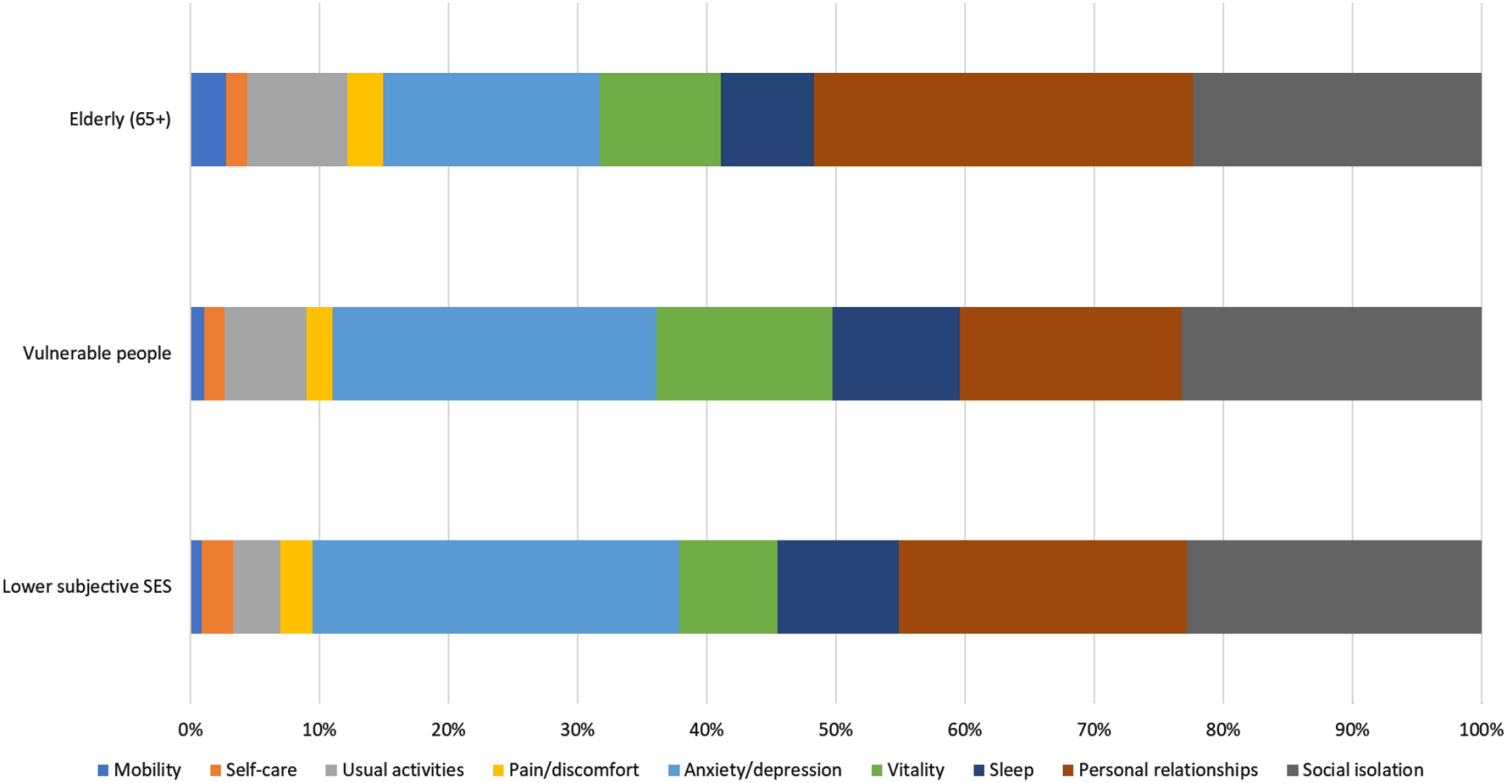
Shorrocks-Shapely decomposition results of global life satisfaction and subgroup analyses results. Elderly (aged 65 and over), 15% total sample. Vulnerable people (lower educated & lower household income), 18% total sample. Lower subjective SES (scored 4 or lower on the MacArthur ladder), 23% total sample

Figure 3 reports subgroup results on SWB. In the elderly group, note the very high relative importance of the two social relationship dimensions, explaining more than half of the total variations in SWB. Also among respondents with low socio-economic status are the two social relationship dimensions relatively more important than in the full sample.

## Discussion

Since the EuroQol Group started its work on which health domains to include in their generic descriptive system, we have seen rising public attention in many countries across the world on the importance of mental health and social isolation—or loneliness, for individuals’ quality of life. Consequently, among health service decision-making agencies that apply EQ-5D, e.g., NICE in the UK, concerns have been raised that these psycho-social aspects are not sufficiently accounted for in the most widely applied generic outcome measure. In response to this, the EuroQol Group has supported the development of a completely new descriptive system, referred to as the EQ health and well-being instrument (EQ-HWB). A large-scale research project sought to identify which items to include, based on a comprehensive literature review and qualitative interviews with patients, social care users, carers, and the general public [28, 29].

As an alternative to developing a brand new instrument, this paper is motivated by an attempt to complement the existing EQ-5D-5L with a set of bolt-ons that fill its psycho-social gap. Further to our previous paper in this journal [14], this paper has provided new and more extensive empirical evidence in support of adding a coherent set of four **p**sycho-**s**ocial bolt-on dimensions, referred to with the acronym PS-4D-4L. Empirical results support the intention of the proposed four bolt-on dimensions to both enrich the measurement of and expand the capability to account for psycho-social health. Among the nine dimensions, the four psycho-social dimensions accounted for 45% of the total explained variations in health-related quality of life (EQ-VAS), and as much as 63% of the total explained variation in well-being (GLS).

Among the four bolt-ons, *Vitality* was by far the most important for EQ-VAS, accounted for 23% of the total explained variation. It is worth noting that this domain is included in all other GPBM (except for Health Utilities Index [30]), as well as being one of the seven key domains in the PROMIS (see Olsen and Misajon [15]). Its importance is likely to reflect the increased attention and prevalence of symptoms like fatigue, exhaustion, and lack of energy [31–36]. *Sleep* was the second most important bolt-on dimension, accounting for 9% of explained variations in EQ-VAS. As part of a descriptive system, sleep is an attractive dimension in that it is a simple word that respondents find easy to comprehend without any more explanations. Furthermore, sleeping problems may stem from various diseases or stress symptoms that are hard to identify by a brief generic descriptive instrument.

The empirical evidence on the importance of adding *Vitality* (or fatigue, tired) and *Sleep* dimensions to EQ-5D is mixed in the literature, depending on which countries have been studied and which methods have been used [37–41]. Nevertheless, these two dimensions are commonly included in the recently developed preference-based measures. Examples include the generic instrument the Child Health Utility 9D (CHU9D) [42] (a comparison with EQ-5D-Y see Chen et al. [43]), the generic PROMIS®-Preference (PROPr) [44] (a comparison with EQ-5D-5L see Klapproth et al. [45]), and the cancer-specific EORTC Quality of Life Utility Measure-Core 10 dimensions (QLU-C10D) [46] (a comparison with EQ-5D-3L see Bulamu et al. [47]).

Lastly, the two social relationships dimensions accounted for 13% of the explained variations in EQ-VAS. *Personal relationships* seek to measure problems with one’s inner circle of family and friends, while *Social isolation* intends to measure problems in the outer circle in terms of community connectedness. While not very important in explaining variations in EQ-VAS, the two dimensions were the most important for well-being; close to 40% of the explained variation in global life satisfaction. Given the increasing empirical evidence on the crucial role of social relationships in health and well-being [16, 48], our results would come as no surprise.

A closer look at the vulnerable populations showed the two social relationships dimensions to be relatively more important for EQ-VAS among individuals with low socio-economic status or position. In contrast, among the elderly (65 +), the two dimensions explained less. This may reflect that as their own physical functioning deteriorates with age, individuals will attend more to the physical health dimensions.

Heterogeneities were also identified for SWB. As compared to the full sample, the two social relationships dimensions were relatively more important in the vulnerable populations considered. The most striking differences were observed in the elderly group, where the two social dimensions accounted for 53% of the total explained variation in SWB. A recent empirical study using more than 1000 older adults (aged 65 and above) in Australia supports the finding of the important role of relationships in SWB based on both revealed and stated preference (discrete choice experiments) data [49].

The main contribution of this paper is to provide strong empirical support for the importance of the four psycho-social dimensions for explaining variations in health and well-being. As an alternative to introducing a brand new health and well-being instrument, there are several advantages of *complementing an existing instrument* with a coherent set of bolt-ons. First, the EQ-5D has achieved a dominant position in applied studies that are designed to measure long-term health outcomes which require that it will be included in future follow-up. Second, the brevity of the descriptive system makes it attractive to include when the total length of the questionnaire is of concern. Third, its current dimensional structure remains relevant for a broad range of diseases. However, for those diseases that have a larger influence on mental and social health, we suggest the current EQ-5D-5L might be complemented with the PS-4D-4L.

Albeit the strong empirical support, it is still premature to combine EQ-5D-5L *and* PS-4D-4L to constitute a new patient-reported outcome measure for use in the real life. As to be discussed in the limitations section, the qualitative study will be a crucial next step to refining the bolt-on classification system and assessing the content validity [50]. To be used as a preference-based measure, a value set that is based on all nine items needs to be developed. It is crucial to further validate the preference-based measure scored using the value set such that the psychometric properties could be reported and this goes beyond the current study (for a preliminary known group validation analysis see Online Supplementary 2).

We acknowledge some limitations in the current study. Although online panels have been widely used in the literature, they are not entirely representative given panel members self-selected to participate in the survey, and they normally represent a higher than average socio-economic group. Further steps have to be solved before this set of bolt-on dimensions is introduced in practice. While the PS-4D-4L is described using terms aligned with the EQ nomenclature, we chose four levels only: no; slight; moderate; and severe problems. The very few respondents ticking level 5 in the current EQ dimensions (0.6% for Self-care, 4.1% for Anxiety/Depression, and 1.0–1.5% for the left 3 dimensions) lean support for disregarding the fifth level. However, we do not have evidence on what proportion of respondents would have opted for level 5 on the bolt-on dimensions if it had been included. Admittedly, more work is needed to test whether to include a fifth level. More generally, there is a need to refine the description of our suggested set of psycho-social bolt-ons, preferably by the involvement of the EuroQol Group. As part of this work, qualitative interviews and focus group discussions with patients and users are required. While we maintain the theoretical and empirical support for the four selected domains, we would welcome more qualitative and psychometric evidence to consider further improvement in their description.

## Supplementary Information

Below is the link to the electronic supplementary material.

Supplementary file1 (PDF 230 KB)

Supplementary file2 (DOCX 26 KB)

## Footnotes

1 According to the length of the duration for each country, the thresholds were set as at least 4 min for respondents from Canada and at least 3 min for respondents from UK or US.

2 Within each country, the following thresholds for gross annual household income were applied: Australia (≤ AU$35,000); Canada (≤ CA$40,000); Norway (≤ 499,000NOK); UK (≤ £20,000); USA (≤ US$30,000).
