# Supplementary material for: Extending the EQ-5D: the case for a complementary set of 4 psycho-social dimensions
Source: Qual Life Res. 2022 Sep 20;32(2):495–505. doi: 10.1007/s11136-022-03243-7 (PMC9486772; doi:10.1007/s11136-022-03243-7)
Supplement: Supplementary file 1 — Supplementary file1 (PDF 230 KB) [file 11136_2022_3243_MOESM1_ESM.pdf]

Quality of Life Research

Extending the EQ-5D: The case for a complementary set of 4 psycho-social dimensions

Gang Chen[1], Jan Abel Olsen[2, 3*]

[1] Centre for Health Economics, Monash Business School, Monash University, Victoria 3145, Australia

[2] Department of Community Medicine, University of Tromsø, 9037 Tromsø, Norway

[3] Division of Health Services, Norwegian Institute of Public Health, 0213 Oslo, Norway

ORCID:

Chen G: 0000-0002-8385-5965

Olsen JA: 0000-0001-9472-2669

[*] Corresponding author:

Professor Jan Abel Olsen, Department of Community Medicine, University of Tromsø, NO 9037 Tromsø,

Tel: +47 4133 2054

Email: jan.abel.olsen@uit.no

Supplementary Material 1 – Additional results

Supplementary Table 1. Correlations among bolt-on dimensions when EQ-5D-5L equals to 1 (N=1,125)

Levels	Vitality	Sleep	Personal relationships	Social isolation
Vitality	1			
Sleep	0.281	1		
Personal relationships	0.186	0.223	1	
Social isolation	0.227	0.219	0.267	1

All Spearman correlation coefficients were statistically significant (all P-value<0.01).

Supplementary Table 2. Exploratory factor analysis

	Factor	
	1	2
[EQ-5D-5L] Mobility	0.917	
[EQ-5D-5L] Usual Activities	0.782	
[EQ-5D-5L] Self-Care	0.696	
[EQ-5D-5L] Pain / Discomfort	0.623	
[EQ-5D-5L] Anxiety / Depression		0.804
[Bolt-on] Social Isolation		0.717
[Bolt-on] Personal Relationships		0.642
[Bolt-on] Sleep		0.568
[Bolt-on] Vitality		0.539

Pattern matrix presented. Extraction method: maximum likelihood. The number of factors was determined by the minimum average partial method. Rotation Method: Oblique Promax (Kappa=4) with Kaiser normalization. Rotated factor correlation: 0.58. Loadings smaller than 0.3 are not shown in the table.

Supplementary Material 2 – Preliminary Known group validation

During the survey with three countries (Canada, the UK, and the US) in April 2020, 5.5% (N=210 out of 3,850 respondents) reported that they were laid off owing to the COVID-19 pandemic. We used this information as a known-group indicator with the hypothesis that those who were laid off would on average have a poor quality of life (Carlsen et al. [2022]).

We created two unweighted summary quality of life scores (ranging from 0 for the worst to 100 for the best quality of life) for the five-dimension EQ-5D-5L and the nine-dimension EQ-5D-5L + PS-4D-4L. First, For each dimension (item), we linearly transferred the original scale onto a 0-100 scale, with 100 indicating no issue and 0 indicating the theoretically most severe issue of each dimension. Next, an average score of relevant dimensions was calculated to produce the overall quality of life score. This unweighted scoring approach is widely used for non-preference-based quality of life instruments, such as the Pediatric Quality of Life Inventory (PedsQL) (https://www.pedsql.org/PedsQL-Scoring.pdf).

Supplementary Figure 1 shows the different distributions of respondents who self-reported being laid off during the survey versus those who did not report being laid off. We could see when using the unweighted summary score of EQ-5D-5L + PS-4D-4L there is a clear impairment in the quality of life of those who were laid off (in Red) whilst the difference is not evident when using the unweighted summary score of EQ-5D-5L alone. The group mean scores (not being laid off vs being laid off) were statistically different when using EQ-5D-5L + PS-4D-4L (80.0 vs 77.8, p=0.004, Mann-Whitney two-sample rank-sum test), whilst insignificant when using EQ-5D-5L (85.5 vs 85.0, p=0.025, Mann-Whitney two-sample rank-sum test).

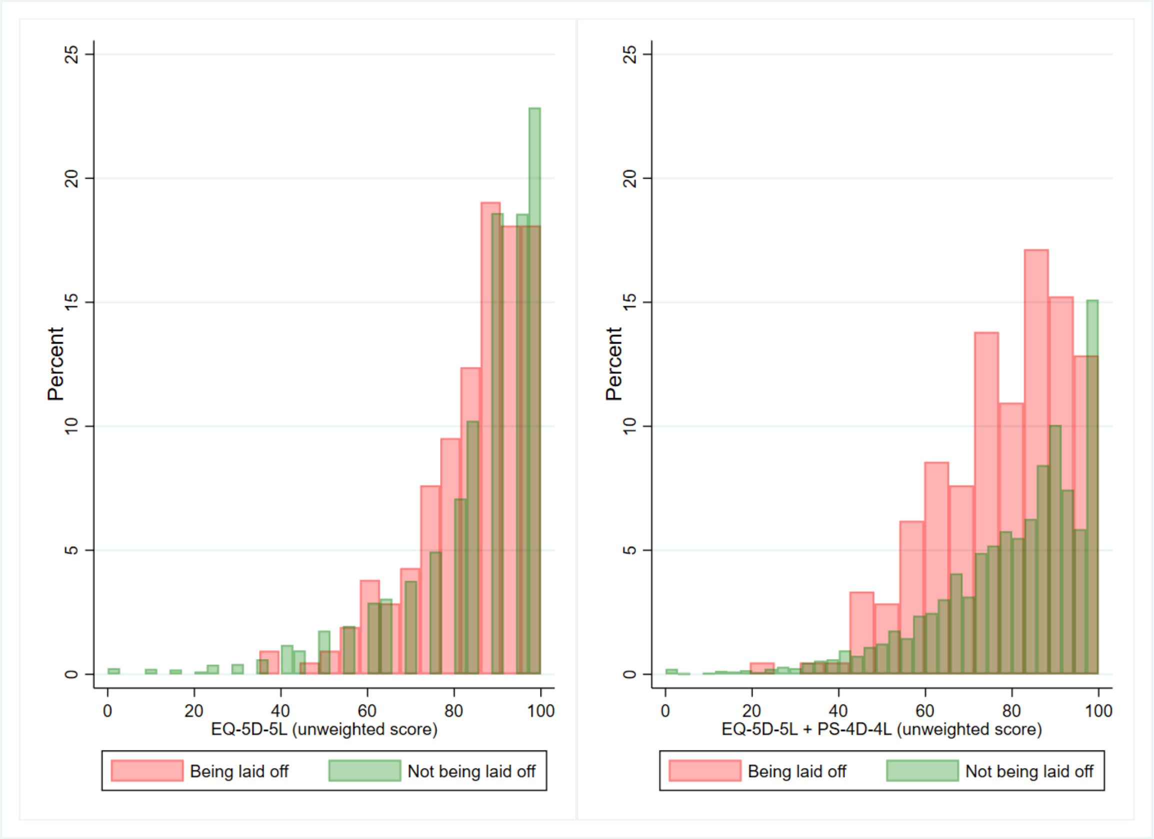

Supplementary Figure 1. Distributions of quality of life scores by measures and whether lay-off status

Reference

Carlsen EØ, Caspersen IH, Ask H, et al. (2022). Association between work situation and life satisfaction during the COVID-19 pandemic: prospective cohort study in Norway. BMJ Open, 12, e049586. DOI: 10.1136/bmjopen-2021-049586.
