# Supplementary material for: Extending the EQ-5D: the case for a complementary set of 4 psycho-social dimensions
Source: Qual Life Res. 2022 Sep 20;32(2):495–505. doi: 10.1007/s11136-022-03243-7 (PMC9486772; doi:10.1007/s11136-022-03243-7)
Supplement: Supplementary file 2 — Supplementary file2 (DOCX 26 KB) [file 11136_2022_3243_MOESM2_ESM.docx]

### Appendix

A description of the four bolt-ons

**Vitality** (feeling energetic)

- I have no problems with lack of energy
- I have slight problems with lack of energy
- I have moderate problems with lack of energy
- I have severe problems with lack of energy

**Sleep**

- I have no problems with sleeping
- I have slight problems with sleeping
- I have moderate problems with sleeping
- I have severe problems with sleeping

**Personal relationships** (family and friends)

- I have no problems with my personal relationships
- I have slight problems with my personal relationships
- I have moderate problems with my personal relationships
- I have severe problems with my personal relationships

**Social isolation** (feeling part of community)

- I have no problems with social isolation
- I have slight problems with social isolation
- I have moderate problems with social isolation
- I have severe problems with social isolation
